# Gut complement C1q and C3 interact with Clostridioides difficile spores via CdeM and contribute to pathogenesis.

**DOI:** 10.64898/2026.09.24.753972

**Published:** 2026-09-25

**Authors:** Marjorie Pizarro-Guajardo, Christian Brito-Silva, Pablo Castro-Cordova, Nicolas Montes-BRavo, Eduardo Callegari, Fernando Gil, Daniel Paredes-Sabja

## Abstract

Clostridioides difficile infection (CDI) is characterized by toxin-mediated epithelial injury, intestinal inflammation, and a high frequency of disease recurrence. Although complement components are produced locally within the gastrointestinal tract, their contribution to C. difficile pathogenesis remains poorly understood. Here, we investigated the interaction of the complement proteins C1q and C3 with C. difficile spores and their contribution to spore-host interactions and disease outcome. We found that C1q and C3 are accessible within the healthy ileal mucosa and that TcdB intoxication differentially remodels their spatial availability, decreasing accessible C1q while increasing accessible C3 at higher toxin concentrations. C. difficile spores associated with both complement components in vivo and directly interacted with purified human C1q and C3 in vitro, with immunogold electron microscopy localizing these interactions to the outer exosporium. Although the collagen-like BclA proteins modulated complement binding under some conditions, they were not required for C1q or C3 association. Far-Western analysis coupled to mass spectrometry identified the exosporium proteins CotE and CdeM as candidate complement-interacting proteins, and purified CdeM inclusion bodies directly associated with both C1q and C3. Exposure of spores to human serum promoted their interaction with intestinal epithelial cells, whereas depletion of either C1q or C3 markedly reduced spore internalization and reconstitution with the corresponding purified protein partially restored entry. C3-derived species, including C3dg, were selectively associated with spores but not vegetative cells, without reducing spore viability. Finally, C1q deficiency did not alter the initial onset of CDI but accelerated recovery and markedly attenuated recurrent disease following vancomycin treatment. Together, these findings identify intestinal complement as a previously unrecognized component of the C. difficile spore-host interface and reveal C1q and C3 as modulators of spore epithelial entry, persistence, and recurrent disease.

## Full Text Availability

The license terms selected by the author(s) for this preprint version do not permit archiving in PMC. The full text is available from the preprint server.

